# Instrumental Music Educators in a COVID Landscape: A Reassertion of Relationality and Connection in Teaching Practice

**DOI:** 10.3389/fpsyg.2020.624717

**Published:** 2021-01-14

**Authors:** Leon R. de Bruin

**Affiliations:** Melbourne Conservatorium of Music, University of Melbourne, Melbourne, VIC, Australia

**Keywords:** music education, pedagogy, phenomenology, epoche, teaching-learning, interpersonal and communication skills

## Abstract

For many countries instrumental music tuition in secondary schools is a ubiquitous event that provides situated and personalized instruction in the learning of an instrument. Opportunities and methods through which teachers operate during the COVID-19 outbreak challenged music educators as to how they taught, engaged, and interacted with students across online platforms, with alarm over aerosol dispersement a major factor in maintaining online instrumental music tuition even as students returned to “normal” face to face classes. This qualitative study investigated the practices employed by instrumental music educators in secondary schools in Melbourne, Australia, analyzing teacher perspectives to music tuition amidst the restriction of interaction with students remotely via online means. Thematic analysis of the interview transcripts revealed music educational approaches that fostered connection, empathy and receptiveness to relationship-building, guiding students in slower and deeper learner-centered approaches, asserting pedagogical practices that reinforced and promoted interpersonal connectedness in and through musical experience and discovery. These findings provide a framework for how music educators can facilitate connection, motivation and student autonomy generating personal meaning and commitment to music making and the learning relationship, which can translate to significant student learning and value in the learning music. Exploring teachers’ pedagogical practices and behaviors within this dyadic teacher-student relationship is a significant addition to the literature, enabling the consideration of the type of connective behaviors required to stimulate and develop long-term interest in music.

## Introduction

Music teachers enter the teaching profession aware of the success rate and attrition of students involved in their studio teaching programs in schools. Instrumental music students – like all students encounter myriad challenges that impact their daily functioning, and positivity to commitment of learning activities. Challenges involve home life, peers, and other aspects of schooling and can have negative effects on students’ social and emotional well-being (Zins et al., 2004). Experienced teacher behavior toward learners provides an attentive, genuine, understanding, and respectful learning relationship reliant on good communication to help children grow emotionally, socially, and academically (Wubbels, 2015). The instrumental music lesson is often a weekly event in students’ lives that promotes aspects of relationship building, motivation, self-autonomy, identity, and community beyond being merely cultivating a better instrumentalist (McPherson and Davidson, 2006; Davidson et al., 2009). In many secondary schools the one-to-one lesson is an enduring construct in the teaching of secondary school musicians and the pedagogical model of preference in bringing student and teacher together in delivering a “serious” instrumental music education (Harrison and Hong, 2004).

In Australia many secondary school students elect to learn an instrument and participate in ensemble activities during and after school hours. Instrumental music tuition in Australian schools in much of 2020 was met with considerable challenge on the part of both teachers and students. Teaching and learning in the State of Victoria in particular required adaptive adjustment and development as the COVID-19 epidemic forced governments to mandate societal isolation requirements that greatly impacted teacher practice. Individual rather than ensemble focus via online communication challenged teacher pedagogy, goal setting, and maintaining of teacher-student connectivity throughout the year. Utilizing constructivist principles that forefront students’ construction, understanding, and experience of learning (Wiggins, 2016), this study explores how instrumental music educators in five secondary schools in Victoria pedagogically supported student learning, and how teachers adapted their teaching, connectivity, and relationality with students over seven months of the 2020 school year through the necessity in teaching instrumental music online for extended periods of 2020.

### Student Engagement

Retention of instrumental students in Australia is an issue with many in the choosing not to continue music beyond middle-school years (Australian Government, 2005; Parliament of Victoria, 2013). Maintaining student engagement in the process of learning and asserting values students perceive in the products of secondary schools’ music programs are an aspect of schools’ music tuition under increased scrutiny (de Bruin, 2018e). Positive engagement in the instrument lesson is a strong determinant in student decisions to continue learning an instrument (Lierse, 2005; Creech and Hallam, 2009), with Lowe (2010) specifically pointing to motivational, cognitive, and emotional benefits significant to retention of instrumental music students in Australian schools.

Student engagement consists of three distinct, yet interrelated, components of students’ commitment and involvement with school and learning, these being behavioral, emotional, and cognitive engagement (Fredricks et al., 2004). Behavioral engagement refers to students’ positive actions, conduct and involvement toward learning. Cognitive engagement spans students’ self-regulated and strategic approaches to learning and the effort to comprehend complex ideas and master difficult skills (Archambault et al., 2009). Thirdly, emotional or affective engagement pertains to students’ sentiments toward school/teachers, senses of happiness, interest, anxiety, and belonging with other students and the teacher. Student engagement is a malleable and developing attribute that varies situationally from one learning situation to another that can be shaped by factors such as features of the classroom and interactions between teachers and students (Fredricks et al., 2004).

### Student Engagement and Teacher–Student Interactions

A critical factor contributing to student engagement is the quality of the teacher–student interactions in the classroom (e.g., Niemiec and Ryan, 2009; Furrer et al., 2014). Allen et al’s. (2013) Teaching Through Interaction (TTI) framework conceptualizes teacher–student interactions through three components: emotional support, classroom organization, and instructional support. These supports focus on ways in which teacher practices foster and facilitate students’ social and emotional functioning (Hamre et al., 2013). Music learning relationships and connectivity have been identified as positive precedents for learning (Gleiser and Danon, 2003; Fischlin and Heble, 2004) with positive behavior support and instructional strategies and feedback crucial in supporting students’ ongoing learning (Hafen et al., 2015). Within the music lesson itself teachers use various collaborative and connective strategies such as scaffolding (Wood et al., 1976), coaching (Schön, 1983), mentoring (Gaunt et al., 2012), and cognitive apprenticeship (de Bruin, 2018b), as well as communicative learning within a “master” and “apprentice” culture (Koopman et al., 2007) to sustain social, emotional, and musical engagement that promotes learning.

Macro-level studies within these strategies have concentrated on music lesson content, management, and the balance between technique and expression (Gaunt, 2006). Within the one-to-one teaching environment Burwell (2012) has investigated prevailing teaching cultures, and (de Bruin, 2017, 2018d) has reported on the significance of interpersonal dimensions in the teacher-student learning relationship. Within this learning domain, salient aspects of teacher interaction point to the influence of verbal interactions on performance behavior (Folkestad, 2005), the impact of verbally prompted behavior involving engaging dialogue (Anderson et al., 2011), inclusive teacher actions that activate participation of students in their learning (Carey and Grant, 2014), and confluence of teacher-student goals and aims (de Bruin, 2018c) pointing to the more refined facets of the inter- and intra-psychological connectivity that promotes how teachers and students come to know “each other’s minds” in the music lesson (Bruner, 1996, p. 12).

Aspects of agency, safety, knowledge, authority and reciprocity reinforce and optimize teacher influence and student motivation and learning. Approaching teaching beyond simplistic notions of knowledge transmission, research has qualified senses of stability (Thelen and Smith, 1998) and sustained affinity between teacher and student (Laible and Thompson, 2007) in developing effective and more enriching learning within a community of musical practice (Lave and Wenger, 1991). Learning relations between instrumental music teachers and their students have been reported to be a conduit toward powerful, effective, and sustainable learning that promotes developmental success, motivation as well as contributing to student well-being (Battistich et al., 1997).

Student-teacher relationships are defined as enduring connections between two individuals, characterized by degrees of continuity, shared history, and interdependent interactions and are a powerful and significant influence on the success of learning (Wentzel, 2012). Gaunt (2008) emphasizes how experienced “teachers are the musical agents, the models, and the motivating forces for their students” (p. 215). However, despite optimal and prolonged moments of synchrony in teacher–student effort the instrumental music lesson is a site of negotiated relations, interactions, behaviors of awareness and focus, frustrations, disappointments, and epiphanies (de Bruin, 2018d). Teacher-student relationships can thus be understood in terms of the interpretations and meanings attributed and derived from moment-to-moment interactions that establish and develop qualities of trust, intimacy, sharing, positive effect, safety, authority, and quality of communication (Kuczynski and Parkin, 2007). These attributes are dynamic, developmental aspects of a learning relationship that need to meet the changing needs of the student over time and across specific contexts (de Bruin, 2018b).

Within the one-to-one lesson, teachers scaffold learning by applying sensitive pedagogical recalibration within “zones of proximal development” (Vygotsky, 1978) in which the teacher moves through a monitor–analyze–assist cycle of interaction. This is an explicit content-related guidance that provides process-related support (Scott, 1998). Dialogic pedagogy (Bakhtin, 1981) and teacher-student interaction in the scaffolding process can enhance a student’s individuality of thought and learning processes through the dialogic positioning *to* and relationships *with* teachers (Matusov and Marjanovic-Shane, 2014). Of added significance to this study is the emphasis on dialogic communication. Talk between teacher and student guides the development of learners’ understandings, creating a contextual experience in which learning, and pedagogy are applied (Mercer and Howe, 2012). This teacher-student collaboration promotes the integrating, elaborating and heighten of students’ adaptability, awareness of and contribution to the learning moment (Rojas-Drummond et al., 2010).

Faced with the prospect of prolonged disruption of face to face lessons, forbidden ensemble-work and diminished connection of students with their peers, teachers sought to emphasize well-being that “maintained the fabric that connects one to others” (Wolf, 2010, p. 130). An important and often overlooked aspect of music education is the reciprocal recognition of the teaching and learning process, knowing that a “bond” exists between teacher and learners in intimate ways, especially given’s music’s (emotional, social, and cultural) potencies (Elliott and Silverman, 2015). Interpersonal teacher-student interactions within the music lesson have revealed elements of “personal chemistry” (Purser, 2005, p. 292), alignments, affinities, tensions, and communication that positively influences learning (de Bruin, 2019).

### The Australian/Victorian Context

The state of Victoria, Australia implemented highly restrictive social measures that included mandatory distancing, stay at home rules and extended school holidays that required Education departments to logistically prescribe how schools would operate. Students in Victoria in 2020 suffered increased levels of stress and anxiety (Russell et al., 2020) with many schools reporting well-being, retention and connection with students being a key driver not just in music education but universally across secondary teaching practice (SEVR, 2020). Students returned to learning through the medium of remote access, with schools using various online media platforms to organize and connect with classes as both teachers and students remained at home yet engaged in teaching and learning. This study explores how instrumental music educators pedagogically engaged and supported student learning through the necessity of engaging solely via online platforms. The research specifically asked, what practices and behaviors facilitated connection and positive learning engagement in music lessons? This question was investigated within a range of cultural and pedagogical contexts that shape the instrumental music educator’s work and diverse secondary school contexts.

## Materials and Methods

The study was framed using Pratt’s (2008) notion of perspectives on teaching where views about teaching and learning both form a part of an ecological perspective of pedagogy. Thus, the research was not focused on the actual impact of teachers’ experiences on their practice but on their perspectives of how their pedagogical teaching and learning practices were adapted to online teaching of instrumental music.

The study was based on interviews with fifteen participant instrumental music teachers across five diverse urban and regional school settings. This included Government and Independent (private- usually religious denominational), Melbourne urban metropolitan as well as a regional city located 1 h north. These schools were selected through purposive sampling (Etikan et al., 2016), and all teachers were equipped with at least 15 years of teaching experience, and each of the 5 schools contained a range of instrumental music teachers (three of brass/woodwind/piano/vocal/percussion). Interviews took place between September 20 and 30, 2020, marking a seven- month period in the state of Victoria of home-to-home isolation and online teaching. Data includes reflections with middle and senior school students (15–18 years of age) of learning relationships ranging from 3 to 6 years in duration. Student’s ability was commensurate with regular, developmental and sustained learning across these experience parameters. Senior students were preparing for external recital examinations.

### Procedure and Analysis

Open-ended interviews were conducted with the participants via zoom and audio recordings transcribed and analyzed. Interviews were conducted in a conversational style based on exploring key ideas raised within the interview (Bhattacharya, 2017). This allowed for adapting the conversation to the experiences of each interviewee and delving deeper to some issues as they arose. This qualitative approach was selected as most apposite, the study situated in the social context of musical teaching, learning and meaning making, that considers the experiences of learning multifaceted, the actions of teachers and learners intentional and within a dynamic process. Each interview began by asking the main research question, the discussion then was tailored to each participant’s experience to allow for deeper exploration. The primary interview questions focused on the interviewee’s teaching experiences in 2020, discussing challenges, constraints and examples of practice and adaptation of practice. As such, the data collected was based on the participants’ reflections of their own practice and beliefs.

This qualitative approach to research emphasized the subjective world of the participant, articulating a “cognitive, meaning-disclosing contribution to what we experience” (Gallagher and Zahavi, 2008, p. 24). It sought to uncover “embodied, experiential meanings” (Finlay, 2009, p. 6) and data that revealed a “richness of thought and purpose” in teachers’ processes (Jorgensen, 2009, p. 7). Hermeneutic (interpretive) phenomenology as a methodology utilizes reflexivity–a person’s reflection upon or examination of a situation or experience that helps interpret meanings discovered. Incorporating a textual Gadamerian hermeneutic of interpreting “texts” of lived experience, the study concentrated on how language reveals being, and the inextricable link between language, understanding, and interpretation (Rapport, 2005).

Multiple readings accompanied by note taking allowed for “hypothetical groupings” that were placed into “tentative pool of categories” (Charmaz, 2003, p. 217). Key words and phrases were then extracted, which were drawn up in a table containing exploratory notes on the side of the manuscript as preliminary interpretations. Emerging themes were then grouped together into general dimensions and placed into hierarchical trees, that were truncated into representative themes that collectively expressed ideas but maintained “the essence of the phenomenon for individual participants’ (Broadbent, 2013, p. 3). These were reduced further, revealing distinctive categories of thematically separated experiences linking experiences together across three dominant themes.

#### Validation and Triangulation Procedures

Procedures included member checking (Lincoln and Guba, 1985) and triangulation procedures were employed in which participants were asked to verify content accuracy prior to the analysis being conducted (Miles and Huberman, 1990). Initial content analysis and meaning unit data were scrutinized in order to ensure that they each contained a single idea and that they had been appropriately named. The arrangement of the meaning units into hierarchies was deliberated throughout the process. Three aspects of triangulation (Reason and Rowan, 1990) included analysis of the description, the intentional interaction, and meaning making. All three were used to discriminate and map participant reflections whilst accommodating the idea that there were emerging possibilities in the analysis of the data. The validity of meaning, understanding, or interpretation of the phenomena was concerned with reaching beyond description, toward deeper cognitive, and experiential explanations. This required a combination of issues considered collectively; the cognitive process of validating data, the subjects’ own perception and the suspending of biases. Utilizing an analytic strategy focusing on inductively interpreted data afforded a rigorous approach to identifying researcher bias and epoché in the findings. Epoché is the processes whereby the researcher sets aside assumptions about phenomenon in question, and where the interpretation of data is conducted “with an open, enquiring attitude” that maintains impartiality and quality (de Bruin, 2021). This was an ongoing researcher consideration throughout the interview and analysis process. The data further reinforced participant voice throughout by the use at times of verbatim quotations and thick description that revealed both the culture and phenomena under analysis (Barrett and Stauffer, 2009).

## Results

Three overarching themes emerged from the data analysis and these were used as the basis for providing a structure for reporting the findings. These themes related to teacher-student adaptability and the need for new encounters, interactive improvisations, and ways of showing and telling. The collaborative nature and co-designed and coordinated aspect of online lessons revealed humanistic qualities of connectivity for student agency that emphasized shared spaces, problem-solving, and relatedness. Another major theme was the dominance of dialogic communication skills. These themes cover aspects of mutual support that helped overcome many of the constraints teachers faced in maintaining connection, engagement and musical improvement in their students.

### Teacher-Student Adaptability

It was apparent that teachers needed to adapt their ways of engaging with students and their methods of interaction. Teachers reflected on their epistemological change in consideration for students’ perceptions of the subject, and how this influenced the pedagogical approaches teachers adopted. Teachers adjusted to accommodate student capacity and receptiveness to modeling, demonstrating and discussion online, self-assessing levels of connectivity and the maintaining of connection with students. This attention to teacher- student communication realized the adoption of more problem-finding approaches, discussing what communicatively was working and what wasn’t. This allowed the “rules of engagement” to be made more visible to students. Forced to interact and rely on a screen for viewing each-other, teachers and students mutually improvised with adaptive approaches to make learning accomplishable. This collaboration required teacher and student to work together as a team to achieve together (Abra and Abra, 1999). Teachers utilized approaches that facilitated connection, discussion, and dialogue, adapting explanation with demonstration. Teachers encouraged students to engage with adjusted and differentiated approaches teachers were adopting, utilizing dialogue, and demonstration by using remote cameras to explicitly show hand/finger/embouchure positioning and manipulation as new “pedagogies of process’ made learning more visible and doable for students (Hattie, 2009). As one teacher stated;

(T3) I found I had to progress more slowly, demonstrate more and allow the students the time to figure things out for themselves. Providing time for students to discuss and verbally acknowledge their learning process allowed students to act more purposefully, more thoughtfully, and with greater awareness of specific accomplishments.

This required teachers to rely on relational capacity and reciprocity with students, based on trust and mutual respect, which enabled honest and open discussion and shared control of the online platform to take place. This provided opportunity for relationships based on “personal control,” and where classroom rules were negotiated and rationale made clear, rather than a “positional control” in which the teacher relied on their position of authority to exert control over students (Bernstein, 2000). Teachers built on this schema of constructing and maintaining positive teacher/student relationships to guide and motivate students, as these teachers expressed:

(T11) Working through an online platform brought our dynamic to a much more equal level. We both needed to hear and see each-other, we both went to extra effort to demonstrate and articulate what we needed to convey. It meant we both had to open up to each other, be less guarded and more conducive to a calm discussion and elaboration of objectives.

Teacher dynamic and class interaction moved from a perspective that teaching involved a monologic classroom discourse to a much more dialogic one. Teachers expressed that as the year progressed, they became more comfortable with teaching online, noting that the students learnt how to talk more succinctly and became more purposeful in their language usage. This teacher commented:

(T4) The first few months involved a lot of working out what’s possible, for both teacher and student. As we became more adept [with online] the students I taught were able to speak with greater clarity of the problems/issues they wanted to talk about in their playing, and we both developed a technical shorthand regarding online technical issues.

This in turn allowed the teachers to realize that students did not need to be led and teachers could trust students to engage in a purposeful and meaningful dialogue of “inter-illumination,” thus allowing for their development as independent learners and active citizens (Fisher, 2007) and as critical thinkers (Mercer, 2000; Lipman, 2003). As this teacher remarked;

(T5) Students became more critical and showed an adaptability that was surprising. We don’t realize the capacities students are capable of, and this predicament has highlighted what they can do given the freedom and compulsion to critically think and problem solve.

The teachers’ changing pedagogical practices allowed for a change in students’ learning too. As teachers changed their practice and adjusted their pedagogical reflexes, this allowed them to see their students as significant critical thinkers and questioners who could enjoy a more active participation in the learning experience (Roche, 2011). Teachers’ beliefs and practices changed and evolved to reflect what Olafson and Schraw (2006) refer to as a contextualist view, one in which students learned through the teacher facilitating a supportive environment where shared understandings could be constructed.

Teachers also changed their views in terms of student learning goals. They noted an improvement in students’ communication of ideas, in participating in online ensemble groups when allowed, an ability to negotiate and reason with others, and in better listening and sharing of the online space when working within a group. As these teachers offered:

(T1) The dynamic between student and I changed, we were there to help each other, to guide each other through and learn from each other.

(T10) This experience was something very new to both students and teachers- and I think we are all changed for the better because of it- we value our relationship more, of working together and making music together.

Teachers saw the value in this heightened connection with students and operating in collaborative and student-centered ways that enhanced a student’s ability to achieve objectives valuable to both the music classroom and elsewhere. As one teacher remarked:

(T2) We created a different learning dynamic – I think it was more involved in the smaller things-more caring but more personable. We got to know each other more deeply, responded to each other more as equals. After a few months, students displayed a confidence in discussing their playing, their learning processes and their reflection on improvement that I tended to overlook in face to face teaching.

Societal and emotional skills can be more clearly defined across qualities of receptiveness and responsiveness to student needs. Empathic relationality and reflexivity play an important role in facilitating and guiding discussion along meaningful lines and in encouraging students to build confidence and self-autonomy (Deci and Ryan, 2000). A set of teacher behaviors identified as effective were the early establishment of ground rules; modeling respect, recognition of difference, critical thinking; and allowing and encouraging students to participate actively (Cotton, 1991). Both teacher and student established a learning situation by adapting, evolving, and exploring possibility thinking in the way they taught and learned (Craft, 2008). Teachers changed their role of teacher to be more a guide, coach, and advisor, encouraging student ownership and empowerment. They instilled a natural discussion and decision-making process and used challenges as opportunities (Elam and Duckenfield, 2000). Several teachers found they had a profound effect on their students’ development, including passion for the subject, social stability, emotional competence, and self-directed learning. This points to a prominent component of instrumental music educators’ jobs in supporting confidence and self-autonomy.

### Dialogic Communication Skills

Dialogic approaches to teaching and learning provided connectivity in student-teacher relationships in terms of approach, teacher/student centeredness, and connection (Wubbels et al., 2006). A dialogic teaching and learning dynamic was discussed by teachers as a means of arranging and organizing ideals and aims, where a thinking together approach could help students and teachers develop an intersubjective understanding and orientation toward one another’s perspectives (Wegerif, 2007). This intersubjectivity – virtual or not, was negotiated between teacher and learner manifested as a perceptual experience that emphasized a shared cognitive understanding and consensus to shaping ideas and enhancing the learning relationship (Spaulding, 2012). Dialogic teaching utilized the power of talk to stimulate and extend students’ thinking and advance their learning and understanding (Alexander, 2004). Dialogue ignited an “in-action” approach that compelled teachers to rethink not just the techniques used to encourage dialogic engagement, but also in the development and, maintenance of flow of ideas and focus in enhancing the way students conceived knowledge.

As Bakhtin (1981) emphasizes, dialogic talk is crucial in classrooms as it is the “inter-illumination” of voices that allows meaning making to occur for students. Teacher’s traditional face to face modes of teaching was “characterized by the familiar rote-learning routines of instruction and recitation…where it is the teacher’s voice that is authoritative and persuasive” (Fisher, 2007, p. 618). Online dialogical pedagogy and student-centered approach are key factors in making learning meaningful for students and recommends that schools and teachers investigate pedagogical practices to allow more of a dialogical approach. A teacher remarked on the amount of talk in lessons;

(T7) I found I talked a lot more with students, and this allowed them to open up and discuss things with me. It was stilted at first but as an unforeseen 8-month experiment, the students are much more articulate, deeper in their reflection and understanding of processes. This is an aspect of my teaching that has – and will continue to change for the better.

Another teacher elaborated on this connective and dialogic pedagogy:

(T3) Through more talk- which was necessary, we found we got to know each other better- I think we responded to each other more receptively, there was more empathy and understanding, where before things were left unsaid- and under-developed.

Teachers discussed dialogic interplay that promoted discussion, critique and argumentation of knowledge and material content. Dialogue was discussed by teachers as spanning aspects of instruction, conversation; and enablement (de Bruin, 2018a). Talk was at times cumulative and formative (Mercer, 2000; Wiliam, 2010) in style. Dialogue occurred via “scaffolding,” “modeling,” and “coaching” procedures (de Bruin, 2018b) that represented a “dynamic exchange of improvised ideas and “flow” [being] generated through the power of the musical interactions’ (St John, 2006). This allowed students to build positively but uncritically on teacher instruction and discussion to find positive ways forward.

Dialogic teaching practices also promoted positive classroom climates by nurturing “supportive teacher-student interactions, good student-student relationships, achievement orientation, and an orderly learning atmosphere” (Vieluf et al., 2012, p. 29). It also involved support for students’ self-determination, which in turn supported autonomy, competence and social relatedness (Ryan and Deci, 2017). Dialogue enabled a cognitive activation and challenge which required deep, challenging content and connectedness between ideas, subjects and real-world contexts (Vieluf et al., 2012). Dialogic teaching required teachers to be more innovative in their pedagogical practices. This involved a process of becoming more aware of student needs and knowing how to connect with them in enacting a more student-centered approach. This teacher explained some benefits:

(T9) I thought it was going to be a wasted year, but we found ways of making successes. Students appreciated being able to talk- their other classes are 25 so instrumental music gave them a voice, and confidence. I got to know them better and I taught better because of this.

Rather than relying on instrumental demonstration and rote modeling and copying, which due to the online method was compromised, teachers and students engaged in thoughtful learning by allowing each other to be active and dialogic participants in the learning process. This allowed a more nurturing teacher-student relationship to prevail, built on trust and reciprocity of understanding that allowed teachers to guide student thinking and action and facilitate the conception of new ideals, goals and creative possibilities. Teachers discussed how they perceived their role as teacher in relation to the student’s learning and how these impacted on how these exchanges influenced the broader macro-culture of the lesson:

(T12) Talking more and opening up possibility for discussion and…openness to elaborate allowed for progress to be made more on the students’ terms. This slowing down actually made more educational sense in allowing students to describe musical sensations and processes. It was a reminder of what should occur more in my teaching, they grew from that sense of connection.

Another said:

I concentrated more on exploring a piece in more detail rather than loading them with technical, solo, and ensemble material. My students could immerse more in the thinking rather than the rote playing of things for the sake of being ready for band. We all enjoyed the freedom to explore aspects of music that we didn’t have time for before isolation.

The teachers implemented a balance between freedom and flexible structures, combining both an improvisatory “feel” and specific design into their dialogic interplay. Teachers asserted a culture of expert practice through interactions that contributed to the growing interpersonal learning relationship. A more student-centered approach were key factors in making learning meaningful for students (c’f. Fisher, 2007; Scholl et al., 2016).

Despite teachers and in most cases, students striving to make best of the online environment, there were repercussions due to the lack of “in class” engagement. Of the fifteen participants, twelve noted that increased numbers had dropped out of music learning. Whilst the pre-COVID face-to-face community facilitated interaction with parents, now communication was available only by email, and required parents to respond in a timely manner. This teacher spoke of the difficulties encountered:

(T11) In some cases, parents were at home, and took an active role in supporting students learning in the lesson. For some students a unique 3-way learning environment developed. However, some students would turn their cameras off- perhaps a habit developed in standard classes. Small groups in my instrumental lessons meant this didn’t happen for very long, but some students chose not to log in, or came into class at progressively late stages of the class. Some were shy about being on camera and being recorded. It was an uphill battle to get parents to enforce engagement- many students were in their rooms and parents had no idea of the disengagement their students were going through. It wasn’t a viable learning experience for some students, and I lost several along the way.

Teachers strived to develop engagement and an increased focus on learning that was meaningful for the learner. Dialogue and relationship building was a key aspect in which learning was inquiry-led and tailored to meet individual learning needs. Teacher and student interactions were more specifically designed to foster and facilitate experimentation in the working out process, teacher relationality, and in students’ learning that promoted focus on collaboration, questioning, and discussion. Whilst this was successful in the majority of cases, there was failure to maintain engagement with students despite the teachers best intentions.

### Connectivity for Student Agency

As well as encouraging a dialogical discourse in the classroom, teacher actions, and engagement with students cultivated collaborative modes of engagement. This involved a pedagogical strategy engaging students in rational questioning and argumentation that allowed learning to be reflected on, articulated, and expressed (Millett and Tapper, 2012). This social and relational approach was what could be referred to as a critical, creative, and caring pedagogy (Lipman, 2003). Its importance resided in the interactive and questioning-based style that brought focus on value-laden questions and discussions that in turn led to better teacher-student and student-student connectivity and relationships (Spooner-Lane et al., 2010).

Adaptive questioning realized additional changes to teachers’ pedagogical practices. The teachers slowed down the pace of delivery as teachers realized the benefits of spending more time enquiring about student learning rather than quickly assuming it and moving on. This allowed time for students to be more purposeful in their language and to articulate their reasoning and thinking behind their answers. This teacher reflected:

(T7) I gave students more time to think and respond; it was a symptom of having to operate online and wait for people to finish speaking, but it gave students more time to think, reflect and talk carefully. With less talking over each other there was more time for students to consider their learning, and more time for me as a teacher to strategize effective approaches.

Teachers responded by describing how this impacted on student confidence. Teachers felt this approach cultivated a supportive learning environment in which students could assert their learning and growing autonomy. Teachers critical approach to engagement promoted students reflecting “through” music (by participating in meaningful inquiries in which they made their own decisions), reflecting “with” music (by developing a deeper understanding of the social situation) and reflecting “on” music (by considering the nature of the subject and how it affected them in their learning and expression of emotion). As one teacher remarked:

(T4) What was missing from the online teaching dynamic and the isolated and detached nature in which students were learning was the sense that students were performing for others. An important aspect of connection was making sure they weren’t just going through the motions, that they were imagining performing for others, or engaging family at home to provide a concert platform. The adrenalin rush we get from performing face to face was an aspect that I needed to recreate in the lesson. Additionally, I saw and became involved with parents being a part of the performance environment much more than before isolation.

Teachers used inherent attributes of music –shared experience, synchrony of musical expression, ideas, and aims to offer students a variety of opportunities to practice many of the skills they need to resolve daily challenges successfully. Despite difficult and challenging circumstances teachers strived to show how personal success was often tied to success with others, and the realization that there are many ways to measure and experience success were examples of skills that teachers perceived of their evaluation of the online experience with students. Teachers reflected on coming to know one’s self better in this situation in order to open spaces for students to be themselves. As one teacher remarked:

(T7) We were all very challenged by these circumstances but forging new ways of operating I found success in approaches I didn’t think would work. And they empowered students’ confidence and determination in ways I didn’t think possible.

The participant instrumental music educators provided students with skills to address challenges. Teachers relational capacity and empathic approaches to constructing learning promoted student agency. Students responded to teachers and the challenges of the new learning environment by themselves being responsive, curious and determined to finding solutions and a way forward.

This brings to focus the recognition that teaching music is a relationally mediated activity. These findings highlight the separation between the requirement to deliver knowledge, but also to motivate students: the two elements do not coincide. It emphasizes that learning music skills and connection to teacher are interwoven so that engagement with activities and the igniting of student passions, motivations and efficacy captures the essence of music education and therefore the deeper function of the teacher.

## Discussion of Findings

These findings provide initial evidence of how instrumental music educators crafted online engagement and learning activity with students. Teachers adapted pedagogically to engage students via an online platform that emphasized aspects of instructional and relational quality impacting on teachers’ perceptions of student development. Teachers created an environment and learning platform in which students felt competent and involved in the tasks undertaken and the knowledge they were able to share. Students were enabled by a strong sense of affiliation and social connectedness to teachers in their activities reflecting Deci and Ryan’s (2000) theory of self-determination involving the satisfaction of specific psychological needs that if satisfied, lead to increased motivation, learning, and well-being.

Perhaps the most important aspect of this study involves the developing relatedness between teacher and student. As social beings, human behavior is dependent on strong forms of connection, social expression, and relatedness. Students’ needs for relatedness encourages the situated learning dynamic that offers safety, shared goals, and ability to express learning in ones’ own terms (Evans et al., 2013). Psychological needs theory posits that learners tend to choose activities that are conducive to success in their social world and reject activities that inhibit it (Deci and Ryan, 2000). Relatedness not just to music but to the music teacher enhanced feelings of connection and identity that was socially, emotionally and environmentally mediated.

This study highlights how relationality and connection are impactful emergent behaviors that activate pivotal teacher and student interactions. Whilst these behaviors rely on an interdependence of elements, it is important to understand the ontology of teacher-student connectivity that promotes an understanding to the precursors, promoters, and prevailers of student learning. Silverman (2020) emphasizes the meaningfulness of embodied, embedded, enacted, and extended accounts of “sense-making” in and for instrumental music learning. Whilst Elliott and Silverman (2015) generalize an “ethic of care” toward students, the COVID context and circumstance of this study details specific qualities and traits teachers responded to enact and qualify this care. Table 1 below details a schematic description and analysis of teacher action in terms of the interactive and relational experience. Initial coding of relational/connective actions discussed by participants in the analysis process delineate the dominant themes of recognition, insightfulness, relatedness, and responsiveness. The secondary and fine-grained analysis of teacher affective and behavioral traits have been further categorized and tabled to outline a graphic spectrum of relational and connective teacher behaviors. Articulating these actions and behaviors is important to the understanding of engagement in a human educative context. It can provide an understanding in the ways in which we as music educators attract students to engage and become involved in their learning, facilitating the learner to find a personalized connection with their teacher and relevance of music. It can bring understanding to teachers regarding the pedagogies and dialogue they use to illuminate learning processes, being attuned to fostering learning through calibration of crafted interactions and learning opportunities.

**TABLE 1:** Relational and connective teacher behaviors.

Recognition	Insightfulness	Relatedness	Responsiveness
Listening	Receptivity	Connectivity	Actioning
Understanding	Imagination	Feeling	Positioning
Comprehension	Evaluation	Sensibility	Reflexing
Awareness	Management	Empathy	Interacting
Apperception	Critique	Predicting	According
Acceptance	Reciprocity	Sensating	Mediating
Perceptivity	Sociability	Postulating	Willingness

This outlining of teacher behaviors highlights aspects of teacher recognition, insightfulness, relatedness and responsiveness that establishes and promotes connection with students. It builds on constructivist principles that suggest cognition functions help learners adapt what we know to different contexts of activity and frame and organize experiences (Dewey, 1933/1958). Teachers as expert practitioners have extensive repertoires of past experiences on which to draw on and respond constructively to problems faced in the instrumental music classroom. Recognition of student needs as a teaching practice is automated or intuitive, shaped by tacitly known knowledge. More sophisticated learning and recognition of student needs is dependent on bringing to consciousness and examining assumptions and values behind actions as teachers.

Students are not merely the objects of their teacher’s behavior; they are animators or co-constructors of their own learning processes. Teachers’ social and emotional connectivity with students provides an emotionally supportive classroom, forms close relationships with students, and foster learners’ social-emotional competence, their emotional limitations and strengths, and how their own emotions impact others (Jennings and Greenberg, 2009). Teacher recognition, awareness, and apperception of learner needs can build on connection and relatedness in developing students independence with novel challenges and new contexts, critically evaluating themselves as learners. Whilst Myhill and Warren (2005) posit that teachers use their instructions to control interactions with their students, teacher perceptivity of student reaction and feedback can promote insights into the social and normative contexts within which students’ learning takes place. For many students the negotiation of “learning how to learn” and the distributed nature self-regulative aspects of planning, action, and reflection and teacher interaction is a dynamic and evolving one (Zimmerman, 2000; de Bruin, 2017). Teacher attentiveness and attunement to promoting not just learning strategies but the learning climate can re-calibrate teacher pedagogy and behaviors that maximize student learning.

## Conclusion

This study investigated instrumental music educators who reflected on their practice to consider how they accommodated learning over 8-month amidst COVID isolation and enforced online engagement. Through qualitative analysis of interview data, connective, communicative, and agentic themes were identified to depict how teachers engaged, promoted, and personalized understanding for student connectivity and well-being. It was clear that teacher participants were passionate about the value of music education, describing diverse experiences and incidents that engaged with and fulfilled musical enrichment for their students. When considering these themes and the ideas inherent to teachers’ educational practices, it is clear that they reflect educator actions to advocate for the fulfillment of four interpersonal qualities that promote connection with teacher and music learning (recognition, insightfulness, relatedness, and responsiveness). It is evident that these participant reflections have illuminated teacher awareness of relational strategies that may continue to work and be successful for teachers and learners once normal face to face environments recommence. With this deeper understanding gained, future research might also examine the levels and qualities of differentiation of connection required by diverse learners as an adept skillset used by teachers. In an age where music education is suffering institutional fiscal constraints and a crowded curriculum, this area of research may pave the way for establishing significant markers of connection, identity, and student self-esteem procured through the unique learning relationships cultivated through music education.

While drawing on a small number of in-depth interviews, these findings speak to the need to understand how to develop a successful and rich secondary school teaching practice. Beyond the scope of this study was the additional work music, emotional involvement and in many cases financial stress and hardship endured by instrumental teachers in ensuring the continuity of instrumental music programs whilst isolated from schools and students. This study shows that emotional support provided by the teacher and organizational support provided through effective ways of engaging and managing learning was beneficial for students’ engagement. It reveals that despite teachers’ striving for connectivity some students refrain from making this connection. The online habits cultivated in a class of 25 plus can be damaging to the high level engagement and interaction that occurs within the instrumental music lesson. Conversely the argument for greater awareness by other subject areas, and indeed Principal/Administrator knowledge of the intense learning and interactions that occur in the instrumental music lesson are qualities that need to be promoted.

These findings highlight the influential role of emotional support on students’ experiences of musical engagement and the relevance of taking into account interpersonal interactions and effects on students’ experiences during lessons. There was a positive relation between teachers’ emotional support and sustained student involvement and progress, which indicate that students tend to be more motivated when they feel they can seek guidance and help from their teachers in emotionally supportive classrooms (Marchand and Skinner, 2007).

This study contributes to literature that general classroom structure to be positively related to aspects of students’ engagement, including emotional engagement (Hospel and Galand, 2016), but specifically indicates that instrumental music classroom organization fosters not only students’ general engagement, but also competence experiences, emotional, and cognitive engagement and well-being. It points to affordances as well as limitations in use recording and reflecting as a pedagogy of practice, where face to face interaction offers the most social and interactive of human qualities and needs. Teachers situational adaptivity, and willingness to themselves explore, improvise and innovate created new learning relations and levels of understanding and reciprocity that allowed students to feel comfortable. Teachers open windows of opportunity to meaningful educational journeys, and those who can demonstrate a rich repertoire of interactional and dialogic teaching skills within diverse and complex systems will be well equipped to meet the needs of the 21st century student.

## Data Availability Statement

The original contributions presented in the study are included in the article/supplementary material, further inquiries can be directed to the corresponding author.

## Ethics Statement

This study was carried out in accordance with the recommendations of the Human Ethics Committee of the University of Melbourne (Ethics ID Number: 2057137.1) with written informed consent from all participants. The patients/participants provided their written informed consent to participate in this study.

## Author Contributions

LB conducted all the research, data gathering, analysis, and writing of this original research project.

## Conflict of Interest

The author declares that the research was conducted in the absence of any commercial or financial relationships that could be construed as a potential conflict of interest.
